# Avoidant/restrictive food intake disorder prognosis and its relation with autism spectrum disorder in Japanese children

**DOI:** 10.1111/ped.70040

**Published:** 2025-05-14

**Authors:** Chie Tanaka, Ayumi Okada, Mana Hanzawa, Chikako Fujii, Yoshie Shigeyasu, Akiko Sugihara, Makiko Horiuchi, Takashi Yorifuji, Hirokazu Tsukahara

**Affiliations:** ^1^ Department of Pediatrics Okayama University Graduate School of Medicine, Dentistry and Pharmaceutical Sciences Okayama Japan; ^2^ Clinical Psychology Section, Department of Medical Support Okayama University Hospital Okayama Japan; ^3^ Department of Child Welfare Notre Dame Seishin University Okayama Japan; ^4^ Department of Epidemiology Okayama University Graduate School of Medicine, Dentistry and Pharmaceutical Sciences Okayama Japan

**Keywords:** autism spectrum disorder, avoidant/restrictive food intake disorder, children, feeding and eating disorders, outcome

## Abstract

**Background:**

There is a lack of reported clinical factors associated with the outcomes of children and adolescents with avoidant/restrictive food intake disorder (ARFID) in Japan. This study aimed to identify these clinical factors and explore the relationship between ARFID and autism spectrum disorder (ASD).

**Methods:**

This retrospective study analyzed data from 48 Japanese children and adolescents with ARFID who visited Okayama University Hospital between January 2011 and March 2022. Clinical characteristics were assessed using medical records and natural history questionnaires. The study compared patients with good and poor prognosis groups and used multiple logistic regression analysis to determine factors influencing prognosis.

**Results:**

The study included 33 patients with good prognoses and 15 with poor prognoses. Comorbid ASD was more prevalent in the poor prognosis group (60%) compared to the good prognosis group (21%). Additionally, more than half of the ARFID patients with comorbid ASD were initially undiagnosed. Multivariate analysis revealed that older age at first visit (*p* = 0.022) and comorbid ASD (*p* = 0.022) were statistically significant factors associated with poor prognosis in ARFID patients. There were no significant differences in body mass index standard deviation score and maximal weight loss between the two groups.

**Conclusions:**

The poor prognosis group had a higher prevalence of comorbid ASD diagnoses. Therefore, it is crucial to evaluate patient's developmental characteristics early in treatment and consider these characteristics throughout the course of care.

## INTRODUCTION

Avoidant/restrictive food intake disorder (ARFID) was officially recognized as a feeding and eating disorder in the Diagnostic and Statistical Manual of Mental Disorders, Fifth Edition (DSM‐5) in 2013.^1^ Patients with ARFID typically exhibit avoidance or restriction of food intake without the presence of body image distortion, a key feature that sets it apart from anorexia nervosa (AN). A previous study noted that picky eating behaviors were present in 28.7% of early childhood ARFID cases.^2^ In Japan, the point prevalence of ARFID‐positive screening among children aged 4–7 years was 1.3%,^3^ while in Switzerland, the incidence of ARFID in children 8–13 years of age was 3.2%.^4^ Notably, ARFID appears to be more prevalent among children with comorbid autism spectrum disorder (ASD).^5^

Globally, the estimated prevalence of ASD stands at 0.76%, showing minimal regional variation.^6^ However, among Japanese children with ARFID, the prevalence of ASD is higher at 12.5%.^7^ Children with ASD are five times more likely to experience feeding difficulties compared to those without ASD.^8^ Research indicates that out of 95 children diagnosed with ASD, 62 (65%) exhibited definite sensory processing problems, which are closely linked to eating problems.^9^, ^10^ Another study reported that children with ARFID had higher levels of food fussiness than those without ARFID.^3^ To the best of our knowledge, the outcomes of ARFID in relation to ASD have not been investigated in the Japanese population.

The primary objective of our study is to evaluate the clinical prognostic factors in patients diagnosed with ARFID. Furthermore, we aim to investigate the prognostic relationship between patients with ARFID and those with comorbid ASD. This information aims to enhance our understanding of the clinical course and presentation of individuals affected by ARFID.

## METHODS

### Participants

This retrospective study conducted at a single center involved 54 Japanese children diagnosed with ARFID at Okayama University Hospital (Okayama, Japan). The inclusion criteria for participants were as follows^1^: children aged 3–18 years diagnosed with ARFID,^2^ their first visit as outpatients was between January 2011 and March 2022, and^3^ at least 12 months have passed since the first visit.

Diagnoses of eating disorders, neurodevelopmental disorders, and psychiatric disorders, not initially identified at the first visit were made using the Diagnostic and Statistical Manual of Mental Disorders, Fifth Edition, Text Revision (DSM‐5‐TR) criteria.^11^ In cases where patients had visited the hospital prior to the adoption of DSM‐5, ARFID diagnosis was retrospectively confirmed by pediatricians specializing in pediatric psychosomatic medicine. ARFID was diagnosed when the severity of the eating disorder surpassed what is typically associated with another condition or disorder, following DSM‐5‐TR guidelines.^11^ Patients lacking follow‐up (*n* = 4) and those with insufficient data for analysis (*n* = 2) were excluded, resulting in a total of 48 patients with ARFID included in the study. Their data with regard to age, sex, body weight, height, body mass index standard deviation score (BMI‐SDS), maximum body weight loss, comorbidities (neurodevelopmental disorder, psychiatric disorder, and other systemic diseases), menstrual history, and school nonattendance history were assessed. All data were available from the medical records and natural history questionnaires. Patients' height and weight were measured at their first visit to our hospital for consultation about eating problems. Menstrual history, encompassing menstruation onset, amenorrhea, and resumption of menses during the study period, was also examined.

Nonattendance at school followed the definition of the Ministry of Education, Culture, Sports, Science and Technology and was defined as the absence from school for ≥30 days in a year because of psychological, emotional, physical, or social reasons other than illness or economic‐related reasons.^12^ Treatment for ARFID patients involved a multidisciplinary approach, including medical management, nutritional rehabilitation, patient education, environmental adjustments (e.g., sharing patient information with schools), pharmacotherapy, meal supervision (adjusting food based on picky eating behavior), supportive psychotherapy, and cognitive behavioral therapy.

The categorization of patients into the good prognosis group encompassed individuals who no longer met the diagnostic criteria for ARFID according to DSM‐5‐TR for at least 3 months by March 2023 or the conclusion of the follow‐up period, based on criteria used in a study comparing the prognosis of AN and ARFID.^13^ Furthermore, this group included patients whose menstrual cycles had resumed post‐menarche or who had reverted to their pre‐ARFID weight growth curve. Conversely, the poor prognosis group consisted of patients who did not meet the criteria for good prognosis or whose condition transitioned to AN.

### Statistical analysis

Continuous variables were expressed as median values and ranges, while categorical variables were presented as frequencies and percentages. Fisher's exact test was utilized for comparing prognosis‐related categorical variables, and the Mann–Whitney U test was employed for continuous variables across both all study participants and those with ASD. Multivariate logistic regression analysis, incorporating statistically significant variables (*p* < 0.05) from univariate analysis along with other clinical factors such as age at first visit, sex, and school nonattendance at first visit, was performed to identify independent prognostic factors for ARFID patients. Adjusted odds ratios, *p* values, and 95% confidence intervals were estimated.

Statistical analyses were conducted using EZR (Saitama Medical Center, Jichi Medical University, Saitama, Japan), a graphical user interface for R (R Foundation for Statistical Computing, Vienna, Austria).^14^ Statistical significance was established at *p* values of < 0.05 for all tests.

### Ethical approval

The research received approval from the Ethics Committee of the Okayama University Graduate School of Medicine, Dentistry, and Pharmaceutical Science and Okayama University Hospital (No. 2206–021). This study was waived of the requirement of a written informed consent from the patients and was conducted in accordance with the Declaration of Helsinki.

## RESULTS

A total of 48 patients were included in this study (Table 1). The median age of the patients was 9.5 years old (range: 4–16 years), with 20 being male. The median BMI‐SDS of the patients was −1.4 (range: −6.1–1.3), and a median maximum body weight loss was 1.6 kg (range: 0–12.0 kg). Nineteen patients had a history of reduced appetite, while seventeen had a history of picky eating. The rate of nonattendance at school was 33% (16/48).

**TABLE 1:** Clinical characteristics of patients with ARFID.

Characteristics	
Patients, *n*	48
Age, years, median (range)	9.5 (4–16)
Sex, *n*	
Male	20 (42%)
Female	28 (58%)
Body weight at first visit, kg, median (range)	24.5 (11.8–52.3)
Height at first visit, cm, median (range)	133.0 (92.0–161.4)
BMI‐SDS at first visit, median (range)	−1.4 (−6.1–1.3)
Maximal amount of weight loss, kg, median (range)	1.6 (0–12.0)
Eating behavior history, *n*	
Having a small appetite	19 (40%)
Picky eating	17 (35%)
School nonattendance at first visit, *n*	16 (33%)
Comorbidity, *n*	
Physical diseases	17 (35%)
Psychiatric disorders	6 (13%)
Neurodevelopmental disorders	18 (38%)
ASD	16 (33%)
Menstrual history (*n* = 28), *n*	
First menstrual period never started	16
Resumption of secondary amenorrhea	3
Secondary amenorrhea	2
Continuous menstruation	7

Abbreviations: ARFID, avoidant/restrictive food intake disorder; ASD, autism spectrum disorder; BMI‐SDS, body mass index standard deviation score.

Among the patients, 17 had comorbid physical conditions, including food allergy (*n* = 3), allergic rhinitis (*n* = 2), bronchial asthma (*n* = 2), brain tumor (*n* = 2), acute lymphoblastic leukemia (*n* = 2), growth hormone deficiency (*n* = 2), irritable bowel syndrome (*n* = 1), orthostatic intolerance (*n* = 1), chronic daily headache (*n* = 1), sleep disorders (*n* = 1), oculocutaneous albinism (*n* = 1), double renal pelvis and ureter (*n* = 1), Osgood‐Schlatter disease (*n* = 1), lymphoma (*n* = 1), hypoplastic left heart syndrome (*n* = 1), and epidermolysis bullosa (*n* = 1). The comorbidities with psychiatric disorders in six cases were as follows: anxiety disorder (*n* = 4), selective mutism (*n* = 2), and adjustment disorder (*n* = 1). The comorbidities with neurodevelopmental disorders in 18 cases were as follows: ASD (*n* = 16, 6 males and 10 females), attention deficit/hyperactivity disorder (ADHD; *n* = 2, 1 male and 1 female), and intellectual disability (*n* = 2). Of the 28 female patients, 16 had not yet experienced menarche.

Thirty‐three patients were categorized into the good prognosis group, while 15 patients were classified into the poor prognosis groups (Table 2). According to the results of univariate analysis, ASD comorbidity (*p* = 0.019) and picky eating (*p* = 0.024) were more prevalent in the poor prognosis group. However, no statistically significant differences were noted in terms of patients' age, sex, BMI‐SDS at first visit, BMI‐SDS at the end of follow‐up, maximal amount of weight loss, nonattendance at school, small appetite history, follow‐up periods, and comorbidities other than ASD (all *p* values > 0.05).

**TABLE 2:** Comparison of prognosis between good and poor outcomes in ARFID.

	Good prognosis	Poor prognosis	*p*
	*n* = 33	*n* = 15	
Age at first visit, years, median (range)	9 (4–16)	10 (6–16)	0.203
Sex, *n*			
Male	15 (45%)	5 (33%)	0.535
BMI‐SDS at first visit, median (range)	−1.4 (−6.1–1.3)	−1.4 (−4.2–0.7)	0.876
BMI‐SDS at the end of follow‐up, median (range)	−1.0 (−2.7–1.6)	−1.8 (−2.8–0.9)	0.128
Maximal amount of weight loss, kg, median (range)	1.5 (0–12)	3 (0–8)	0.424
Eating behavior history, *n*			
Having a small appetite	12 (36%)	7 (47%)	0.538
Comorbidity, ASD	3 (25%)	5 (71%)	
Picky eating	8 (24%)	9 (60%)	0.024*
Comorbidity, ASD	4 (50%)	6 (66%)	
School nonattendance at first visit, *n*	10 (30%)	6 (40%)	0.527
Comorbidity, *n*			
Neurodevelopmental disorders			
ASD	7 (21%)	9 (60%)	0.019*
ADHD	1 (3%)	1 (7%)	0.532
Intellectual disability	2 (6%)	0 (0%)	1
Psychiatric disorders			
Selective mutism	0 (0%)	2 (13%)	0.090
Adjustment disorder	1 (3%)	0 (0%)	1
Anxiety disorder	2 (6%)	2 (13%)	0.592
Physical disorders	14 (42%)	3 (20%)	0.196
Follow‐up periods, months, median (range)	27 (1–126)	21 (1–95)	0.332
Clinical course of ARFID, *n*			
Completed treatment after remission	18 (55%)		
Follow‐up post‐remission	3 (9%)		
Continuous consultation post‐remission	9 (27%)		
Transfer to another hospital post‐remission	3 (9%)		
Continuous treatment		8 (53%)	
Interrupted treatment		4 (27%)	
Transferred to another hospital for exacerbation ARFID		2 (13%)	
Changed disease type (AN)		1 (7%)	

Abbreviations: ADHD, attention deficit/hyperactivity disorder; AN, anorexia nervosa; ARFID, avoidant/restrictive food intake disorder; ASD, autism spectrum disorder; BMI‐SDS, body mass index standard deviation score.

* *p* < 0.05.

The details of the outcomes are as follows: among the 33 patients in the good prognosis group, 18 (55%) completed treatment after achieving remission, 3 (9%) underwent follow‐up post‐remission, 9 (27%) continued with consultations post‐remission, and 3 (9%) transferred to a different hospital post‐remission. By contrast, among the 15 patients in the poor prognosis group, 8 (53%) received continuous treatment, 4 (27%) experienced interrupted treatment, 2 (13%) transferred to a different hospital for worsened ARFID, and 1 (7%) saw a change in diagnosis to AN.

Of the patients with ARFID and comorbid ASD, 57% (4/7) of those in the good prognosis group and 56% (5/9) of those in the poor prognosis group were initially not diagnosed with ASD during their first visit. No statistically significant differences were observed between the prognostic groups of ARFID patients with ASD regarding age, sex, BMI‐SDS at first visit, BMI‐SDS at the end of follow‐up, maximal amount of weight loss, nonattendance at school, small appetite history, and picky eating history (all *p* values > 0.05) (Table 3).

**TABLE 3:** Specifics of ASD comorbidity.

	Good prognosis *n* = 7	Poor prognosis *n* = 9	*p*
Age at first visit, years, median (range)	8 (4–10)	9 (6–14)	0.239
Sex, *n*			
Male	3 (43%)	3 (33%)	1
Female	4 (57%)	6 (67%)	
Maximal amount of weight loss, kg, median (range)	0.7 (0–5)	3.2 (0–5)	0.242
BMI‐SDS at first visit, median (range)	−1.3 (−2.1–0.8)	−1.4 (−4.2–0.7)	0.958
BMI‐SDS at the end of follow‐up, median (range)	−0.9 (−1.4–0.4)	−2.2 (−2.6–0.1)	0.252
No diagnosis of ASD at first visit, *n*	4 (57%)	5 (56%)	1
Having a small appetite, *n*	3 (43%)	5 (56%)	1
Picky eating, *n*	4 (57%)	6 (67%)	1
School nonattendance, *n*	2 (29%)	4 (44%)	0.633

Abbreviations: ASD, autism spectrum disorder; BMI‐SDS, body mass index standard deviation score.

Upon multivariate analysis, older age at first visit (odds ratio 1.42; 95% confidence interval 1.05–1.93; *p* = 0.022) and comorbid ASD (odds ratio 8.01; 95% confidence interval 1.35–47.40; *p* = 0.022) emerged as statistically significant factors with poor prognosis in patients with ARFID compared to other factors such as sex, school nonattendance at first visit and picky eating (Table 4).

**TABLE 4:** Multivariate analysis examining prognostic factors in ARFID.

Factors (poor prognosis = 1)	OR	95% CI	*p*
Age at first visit (per 1 year increase)	1.42	1.05–1.93	0.022*
Sex (male = 1)	0.80	0.16–4.02	0.790
Comorbidity: ASD (positive = 1)	8.01	1.35–47.40	0.022*
School nonattendance at first visit (positive = 1)	1.27	0.25–6.40	0.774
Picky eating (positive = 1)	3.79	0.76–18.90	0.104

Abbreviations: ARFID, avoidant/restrictive food intake disorder; ASD, autism spectrum disorder; CI, confidence interval; OR, odds ratio.

* *p* < 0.05.

## DISCUSSION

Our research findings indicate a significant association between comorbid ASD and a history of picky eating in patients with ARFID, suggesting a poorer prognosis based on univariate analysis. Additionally, multivariate analysis revealed a significant association between ASD comorbidity and age at first visit in ARFID patients, further indicating a poor prognosis. Older age at first visit was associated with poorer prognosis.

Dumont et al. from Netherland revealed that sex (male), syndrome/intellectual disability, and a lack of varied nutritional intake were predictors of a worse prognosis in patients with ARFID who had a history of behavioral day treatment.^15^ They also reported that ASD comorbidity was not a statistically significant worse prognosis predictor. Inconsistent with the findings of their study, our study did not identify gender or comorbid intellectual disability as factors contributing to poor prognosis. Their study suggested that many patients were young and had genetic disorders or intellectual disabilities. It can be suggested that the difference of participants affected the results. Additionally, our study showed a higher prevalence of ASD compared to other reports, and comorbid sensory hypersensitivity in children with ASD may also account for the differences.

Moreover, a prior study included patients with ARFID who had a history of picky eating since early childhood (28 out of 98 patients).^2^ By contrast, our study revealed that 17 of 48 patients had a history of picky eating, which was more prevalent in the poor prognosis group. Picky eating may stem from a lack of diverse nutritional intake, a factor associated with poor prognosis according to Dumont et al.^15^ The inclusion of age at first visit as a factor in poor prognosis may be attributed to prolonged picky eating since early childhood. ASD is commonly characterized by persistent deficits in social communication and restricted, repetitive interests.^1^ Children with ASD often exhibit specific eating behaviors such as reluctance to try new foods, limited interest in new foods, and reduced food intake during periods of emotional distress like anger, fatigue, or sadness.^16^ These eating patterns are influenced by the characteristics of ASD and may impact the duration and effectiveness of ARFID treatments in affected individuals.

It is noteworthy that a significant proportion of ARFID patients are diagnosed with ASD only after being diagnosed with ARFID, highlighting the importance of assessing developmental characteristics early in the treatment process. There are two main reasons why ASD may not be detected early and is often diagnosed later: First, as per DSM‐5 guidelines, ASD symptoms are usually present from an early developmental stage but may not fully appear until social demands surpass the child's abilities. Increased psychosocial stress in school and group settings may then reveal these developmental characteristics and lead to a delayed ASD diagnosis. Second, parents and others may attribute picky eating and small meals to “personality,” which can delay seeking specialist consultation, potentially resulting in long‐term nutritional deficiencies and negative impacts on neurodevelopment.^17^ Therefore, early detection of ASD is crucial.

It was suggested that a higher BMI in patients with AN predicted better outcome at the end of treatment and that higher weight influenced the positive impact on prognosis across all levels of care.^18^ However, in our patient cohort, there were no discernible differences in BMI‐SDS and weight loss between the ARFID groups classified as having good and poor prognoses. This observation may be attributed to the inclusion of patients with varying disease onsets, encompassing those who experienced rapid weight loss as well as those with long‐term poor weight gain. Our findings suggest that neither BMI‐SDS nor the degree of weight loss could reliably indicate disease severity or influence prognosis. Although we could not examine this due to insufficient data, early weight gain following the start of treatment has been associated with a good prognosis.^19^ Therefore, improving nutritional status as soon as possible after diagnosing ARFID is crucial.

Additionally, there was no statistical difference in the proportion of children with school nonattendance at their first visit between the two groups. However, it is important to acknowledge that reduced opportunities for interaction with school personnel and other nonfamily members may lead to delayed detection of ARFID.

This study possesses several limitations. First, due to the lack of uniformity in the treatment approaches utilized, the evaluation of treatment effects may be insufficient. Second, being a retrospective single center study increases the potential for selection bias in case inclusion (e.g., concurrent medical condition). Furthermore, the retrospective design limited our ability to fully evaluate patient characteristics like food preferences and sensory processing issues. The relatively small sample size also restricts the identification of other potential variables linked to poor outcomes in ARFID patients. Therefore, larger prospective multicenter studies are needed to thoroughly investigate the prognostic value of various patient characteristics in ARFID.

## CONCLUSIONS

The findings of this study reveal that individuals in the poor prognosis group of ARFID exhibited a higher prevalence of concurrent ASD compared to those in the good prognosis group, with approximately half of the ASD cases remaining undiagnosed at the onset of treatment. This underscores the importance of early assessment of patients' developmental characteristics and their consideration throughout the treatment process.

## AUTHOR CONTRIBUTIONS

Chie Tanaka, Ayumi Okada, Yoshie Shigeyasu, Chikako Fujii, Akiko Sugihara, and Mana Hanzawa. were involved in the conception and design of the study. Data collection was carried out by Chie Tanaka, Ayumi Okada, Yoshie Shigeyasu, Chikako Fujii, and Akiko Sugihara, Takashi Yorifuji supervised the statistical analysis. Chie Tanaka wrote the first draft of the manuscript, and Ayumi Okada, Makiko Horiuchi and Hirokazu Tsukahara revised it critically for important intellectual content. All authors contributed to the interpretation of data, drafting and revision of the manuscript, and approved the final version of the manuscript.

## CONFLICT OF INTEREST STATEMENT

The authors declare no conflict of interest.
